# Development of a Whole-Cell System Based on the Use of Genetically Modified Protoplasts to Detect Nickel Ions in Food Matrices

**DOI:** 10.3390/ijms25116090

**Published:** 2024-05-31

**Authors:** Monica De Caroli, Carla Perrotta, Patrizia Rampino

**Affiliations:** 1 Department of Biological and Environmental Sciences and Technologies, University of Salento, Via Monteroni 165, 73100 Lecce, Italy; monica.decaroli@unisalento.it (M.D.C.); carla.perrotta@unisalento.it (C.P.); 2 NBFC National Biodiversity Future Center, 90133 Palermo, Italy

**Keywords:** whole-cell system, *Nicotiana tabacum* protoplasts, *HSP* promoter, heavy metals, food safety

## Abstract

Heavy metals are dangerous contaminants that constitute a threat to human health because they persist in soils and are easily transferred into the food chain, causing damage to human health. Among heavy metals, nickel appears to be one of the most dangerous, being responsible for different disorders. Public health protection requires nickel detection in the environment and food chains. Biosensors represent simple, rapid, and sensitive methods for detecting nickel contamination. In this paper, we report on the setting up a whole-cell-based system, in which protoplasts, obtained from *Nicotiana tabacum* leaves, were used as transducers to detect the presence of heavy metal ions and, in particular, nickel ions. Protoplasts were genetically modified with a plasmid containing the *Green Fluorescent Protein* reporter gene (*GFP*) under control of the promoter region of a sunflower gene coding for a small Heat Shock Protein (HSP). Using this device, the presence of heavy metal ions was detected. Thus, the possibility of using this whole-cell system as a novel tool to detect the presence of nickel ions in food matrices was assessed.

## 1. Introduction

Heavy metals are considered the most dangerous contaminants for the environment; they constitute a threat to human health, even when present in traces. The principal sources of heavy metal are the discharge of industrial effluents and fertilizers, which are responsible for soil contamination. The main threat to human health derives from the capability of metal ions to persist in soils, where they tend to accumulate and are easily transferred into the food chain [1,2], causing damage to human health, such as toxicity and carcinogenicity. The ingestion of heavy metal-contaminated vegetables can cause serious health problems, such as malnutrition, gastrointestinal cancer, the depletion of essential nutrients, the weakening of the immune defense, and the inhibition of mental growth (which also occurs during intrauterine development) [3,4]. Although it has been assessed that the presence of some heavy metals in trace amounts is nutritionally essential for healthy life, they can become toxic when they accumulate in human soft tissues because they are not metabolized. This causes a decrease in energy levels in vital organs, changes blood composition, and reduces mental and central nervous function [5].

Among heavy metals, nickel appears to be one of the most dangerous because it is naturally present in drinking water as well as in many food matrices, exposing the population ingesting it. Sensitivity to nickel prevalence varies in different countries, sitting in a range between 4 and 13.1% [6,7,8]. Nickel is extremely harmful for health because it can cause different disorders such as kidney, lung, and cardiovascular diseases, dermatitis and, sometimes, even some kind of cancer [9]. Therefore, public health protection certainly requires nickel detection in the environment and food chains. At present, the existing techniques used for trace analysis of heavy metals include chromatographic, voltametric, and spectroscopic methods. However, all these methods are quite expensive and are difficult to use for in situ analysis (an analysis conducted directly on the location where the sample is found). On the contrary, new tools such as biosensors can be used as simple, rapid, and sensitive methods to detect heavy metal contaminants, also performed using in situ analyses.

Classical biosensors are analytical devices characterized by three elements: a biological recognition element; a physical–chemical transducer converting the biological response into a detectable signal; a micro-electronic component able to amplify and convert the signal into a numeric record [10]. In particular, when a prokaryotic or eukaryotic cell represents a reporter system that incorporates both biological recognition and transducer elements, the device is called “whole-cell biosensor” or, in other words, it constitutes a whole-cell detection system [5,11,12]. This kind of biosensor responds to the presence of contaminants or to physiological stresses, producing a detectable cellular output signal. Generally, the cells used as biosensors are engineered to acquire the ability to behave as transducers or to amplify their sensitivity by introducing reporter genes, controlled by promoters that respond to environmental stimuli. Most of the cell types considered particularly useful for metal ion detection are genetically modified bacteria, although eukaryotic cells, such as yeast, algae, or protozoan, can also be used [5].

In this paper, we report setting up a whole-cell-based system, in which protoplasts obtained from *Nicotiana tabacum* leaves were used as transducers to detect the presence of heavy metal ions. The whole-cell detection system was based on the ability of plant cells to respond to environmental abiotic stresses such as the presence of metal ions, which elicits a molecular response. *N. tabacum* protoplasts were genetically modified with a plasmid containing the *GFP* reporter gene under the control of the promoter region of a sunflower gene coding a small heat shock protein (HSP). This device was used to test fro the presence of nickel ions in different food matrices known to possess high nickel contents, exploring the possibility of using this biosensor as a novel tool to detect the presence of nickel ions in food matrices.

## 2. Results

### 2.1. Protoplasts Transformation and Immobilization

**Figure 1:**
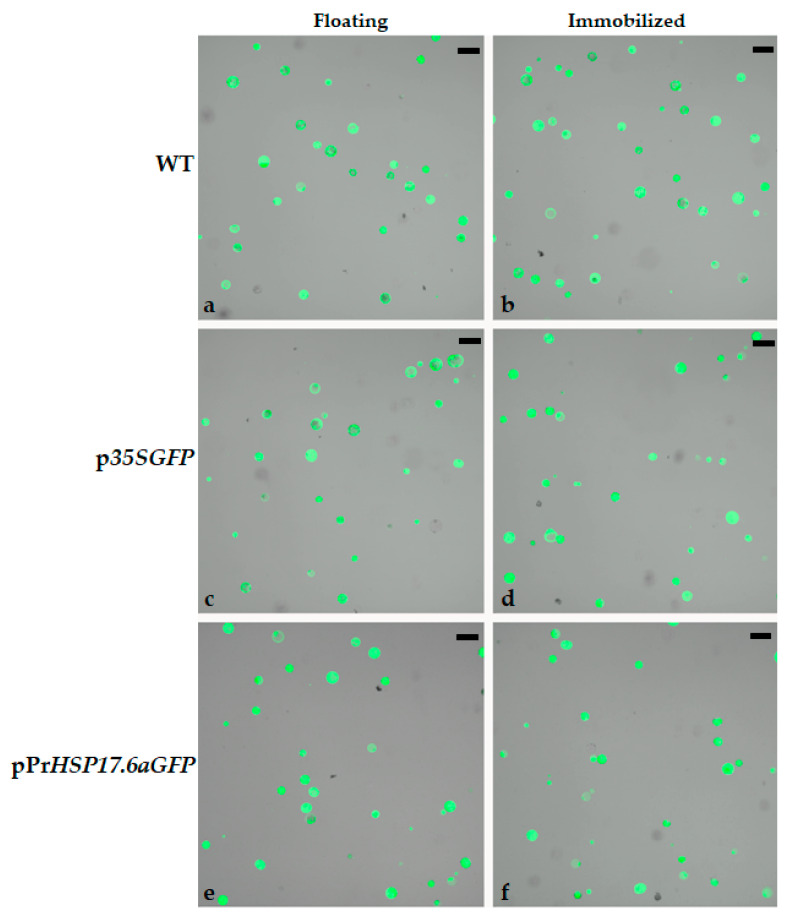
Confocal microscope images of tobacco protoplasts after FDA staining. WT—untransformed protoplasts; p*35SGFP*—protoplast transformed with p*35SGFP* plasmid; pPr*HSP17.6aGFP*—protoplast transformed with pPr*HSP17.6aGFP* plasmid. (**a**,**c**,**e**) Floating protoplasts; (**b**,**d**,**f**) protoplasts immobilized in K3 medium containing 0.6% agarose. Scale bars: 50 µm. Objective: 10×; zoom: 0.6.

Protoplasts obtained from leaves of *N. tabacum* were transformed with p*35SGFP* or pPr*HSP17.6aGFP* plasmids, containing *GFP* genes, under the control of the constitutive *CAMV35S* or the inducible sunflower *HSP17.6a* promoters, respectively. Protoplasts were in part maintained in a liquid K3 medium (floating) and in part immobilized in a K3 medium containing agarose (0.6%), being distributed into 96 multi-well plates. Untransformed (WT) and transformed protoplasts were tested for their viability by using a fluorescein diacetate (FDA) assay. FDA is a fluorophore that is able to penetrate living and dead cells, where it makes only the viable cells fluorescent; in fact, the fluorescent labelling is due to fluorescein cleavage by cellular esterases, which are only active in viable cells [13], promoting the emission of green fluorescence [14]. Untransformed (WT) and transformed protoplasts were observed using a confocal microscope. Protoplasts images, as seen in Figure 1, indicate that floating and immobilization do not alter the structure and functionality of protoplats, as deduced by the spherical form of fluorescent cells. Further, the number of viable protoplasts was determined. Table 1 reports that the percentage of viable protoplasts is almost the same for WT and transformed protoplasts, as well as for floating and immobilized protoplasts. Therefore, we deduced that our protoplast system was suitable for further analyses.

**Figure 2:**
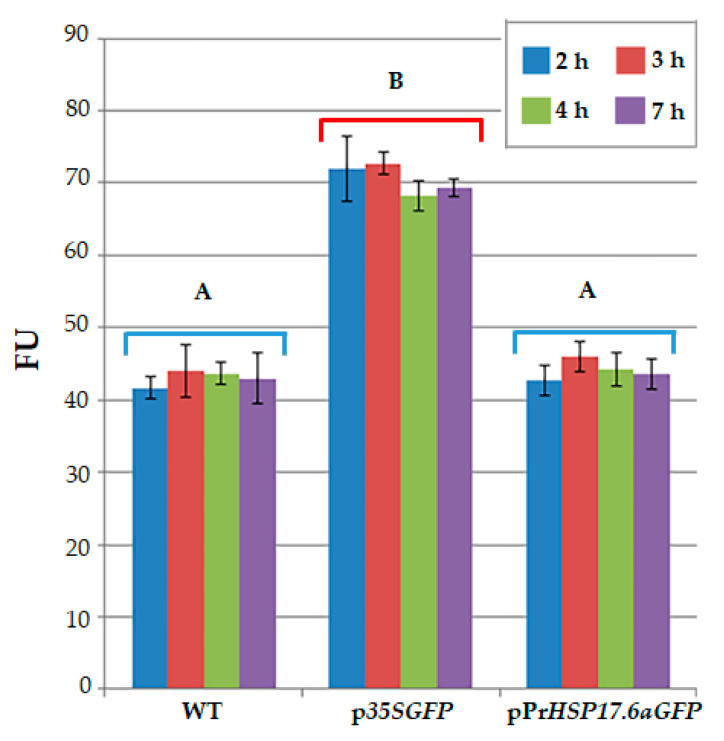
Evaluation of fluorescence, measured by a fluorometer, after 2, 3, 4, and 7 h from immobilization of untransformed protoplasts (WT), protoplasts transformed with p*35SGFP* plasmid (p*35SGFP*), and protoplasts transformed with pPr*HSP17.6aGFP* plasmid (pPr*HSP17.6aGFP*). Each value represents the mean of three independent measurements ± SD. Different uppercase letters indicate significant differences among the FU values (*p* < 0.001).

Immobilized protoplasts were analyzed to determine auto-fluorescence emissions. Fluorescence was measured in wild-type, as well as in engineered protoplasts, after 2, 3, 4, and 7 h from immobilization. A fluorescent signal was detected for all the protoplast samples. As reported in Figure 2, the level of the signal is almost the same during the time spent (after 2 to 7 h) on each group of protoplasts. Moreover, as expected, the fluorescence signal detected for p*35SGFP*-transformed protoplasts is always higher than the fluorescence signal detected for untransformed protoplasts (WT), as well as for pPr*HSP17.6aGFP*-transformed protoplasts. Statistical analysis indicated that there is no statistically significant difference in fluorescence signaling, measured at the various time points and expressed as fluorescence units (FU), within each protoplast group (WT, p*35SGFP* and pPr*HSP17.6aGFP*-transformed protoplasts); moreover, no significant difference was observed between the WT and pPr*HSP17.6aGFP*-transformed protoplast groups. On the contrary, a highly significant difference (*p* < 0.001) was observed between the p*35SGFP*-transformed protoplast groups and the WT group or the pPr*HSP17.6aGFP*-transformed protoplast groups (Figure 2).

**Figure 3:**
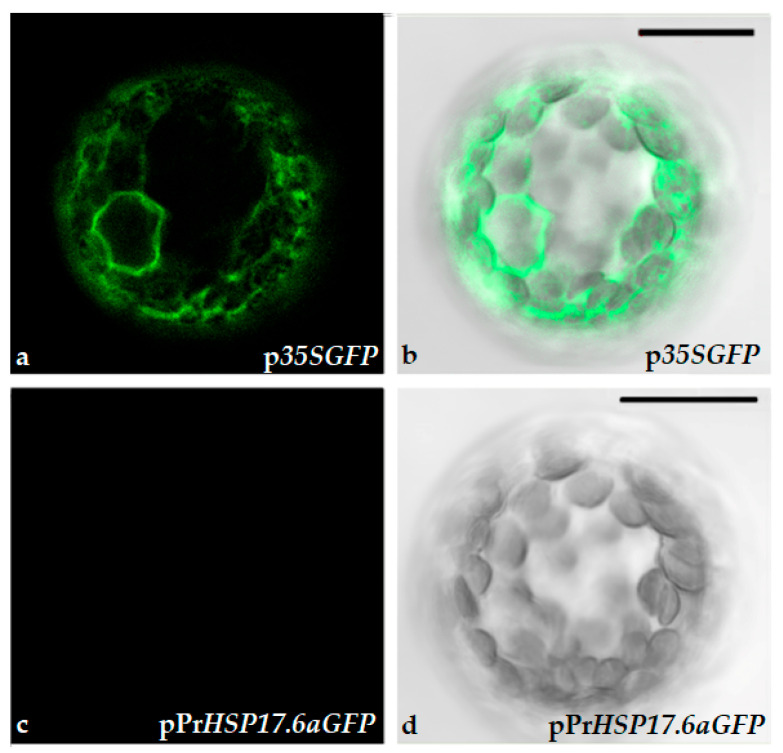
Confocal microscope images of immobilized tobacco protoplasts. (**a**,**b**) Protoplasts transformed with p*35SGFP* plasmid; (**c**,**d**) protoplasts transformed with pPr*HSP17.6aGFP* plasmid. (**b**,**d**) Bright field. Scale bars: 20 µm. Objective 40×, zoom 2.0.

To verify that differences in fluorescence were due to *GFP* gene expression, driven by the *CAM35S* constitutive promoter, protoplasts were observed via confocal microscopy. The data obtained indicated that differences in fluorescence, detected by the fluorometer, were due to the expression of GFP since only p*35SGFP*-transformed protoplasts exhibited green fluorescence (Figure 3).

### 2.2. Responsiveness of Engineered Protoplast to Heavy Metal Ions

**Figure 4:**
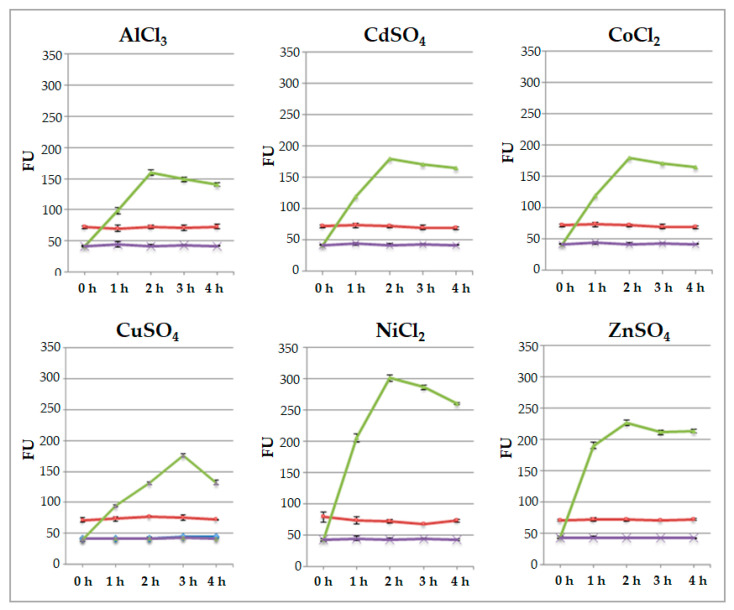
Evaluation of fluorescence by fluorometer after 1, 2, 3 and 4 h from protoplast immobilization. Red line: protoplasts transformed with p*35SGFP* plasmid; violet line: untreated protoplasts transformed with pPr*HSP17.6aGFP* plasmid; and green line: protoplasts transformed with pPr*HSP17.6aGFP* plasmid plus metal ion solutions (50 μL of 20 μM each). Each value represents the mean of three independent measurements ± SD.

To test the ability of engineered protoplasts to sense the presence of metal ions, immobilized protoplasts were added with 50 μL of 20 μM each AlCl_3_, CdSO_4_, CoCl_2_, CuSO_4_, NiCl_2_, and ZnSO_4_ at room temperature. Fluorescence was measured by a fluorometer after 1, 2, 3, and 4 h. The values of fluorescence were measured in protoplasts transformed with p*35SGFP* plasmid, used as the control, as well as in protoplasts transformed with pPr*HSP17.6aGFP* plasmid. The values detected are shown in Figure 4 and indicate that the signals of p*35SGFP*-transformed protoplasts remain unchanged during the time course of all salt treatments. The fluorescence signals of pPr*HSP17.6aGFP*-transformed protoplasts increased during time course, reaching a maximum level after 2 h and remaining almost the same afterwards. When CuSO_4_ was used, and only then, the maximum induction was reached after 3 h and the fluorescence signal declined thereafter.

**Figure 5:**
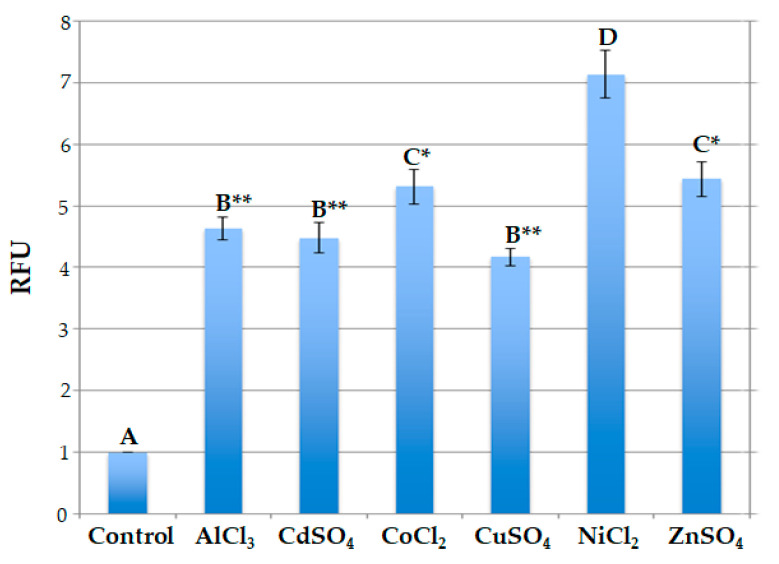
Relative fluorescence (expressed as relative fluorescence units, RFUs) at the maximum level of detection of protoplasts transformed with pPr*HSP17.6aGFP* and treated with different metal ions (50 μL of 20 μM each). Each value represents the mean of three independent measurements ± SD. Uppercase letters indicate statistically different values among the various protoplast groups. Asterisks correspond to statistically different values between the NiCl_2_-treated protoplasts and each other different group (*, significant difference, *p* < 0.05; **, highly significant difference, *p* < 0.001).

Moreover, the data obtained indicate that the signal’s intensity depends on the specific metal ion added. In particular, after 2 h, in the case of NiCl_2_ treatment, the signal was over 7-fold higher than that of the untreated protoplasts; it was more or less 5.5-fold higher after treatment with ZnSO_4_ and CoCl_2_; and it was 4.5-fold higher when using AlCl_3_ and CdSO_4_ salts. Following CuSO_4_ treatment, the maximum value was 4-fold higher (Figure 5).

Comparison among the relative fluorescence values displayed by engineered protoplasts indicated that these values were similar when engineered protoplasts were treated with AlCl_3_ and CdSO_4_ or with CuSO_4_; in fact, in these cases, no statistically significant difference was observed in terms of relative fluorescence values. The fluorescence signals were also almost the same in the presence of CoCl_2_ and ZnSO_4,_ and no statistically significant difference was observed in this case. On the contrary, statistically significant differences were observed when the florescence signal values of protoplasts treated with Ni ions were compared with those of all the other value groups. In particular, highly significant differences (*p* < 0.001) were observed when the values reached by protoplasts treated with Ni ions were compared to the ones obtained after treatment with Al, Cd and Cu ions, while significant differences (*p* < 0.05) were observed when they were compared to the ones obtained after treatment with Co and Zn ions (Figure 5).

**Figure 6:**
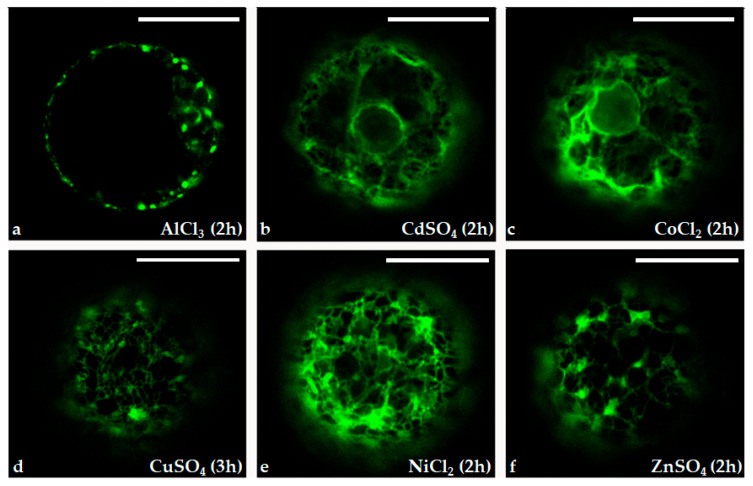
Confocal microscope images of the immobilized protoplasts transformed with pPr*HSP17.6aGFP* plasmid in presence of 50 μL of 20 μM AlCl_3_ (**a**), CdSO_4_ (**b**), CoCl_2_ (**c**), CuSO_4_ (**d**), NiCl_2_ (**e**) and ZnSO_4_ (**f**). Scale bars: 20 µm. Objective: 40×; zoom: 2.0.

These data were confirmed via confocal observations and the fluorescence quantification of the protoplasts treated with the six different heavy metal ions (Figure 6). The protoplasts tested with all heavy metal ion treatments appeared to be fluorescent, although they showed different intensity and fluorescence patterns. The compartments of the secretion pathway (nuclear membrane in continuity with the endoplasmic reticulum and the Golgi complex) were clear in the protoplasts characterized by a higher level of GFP expression, i.e., protoplast treated with 50 μL of 20 μM CoCl_2,_ NiCl_2,_ and ZnSO_4_.

These data were also confirmed by fluorescence index values (Table 2). A comparison of the fluorescence index values obtained after NiCl_2_ treatment indicates significant differences with respect to all other salt treatments.

**Figure 7:**
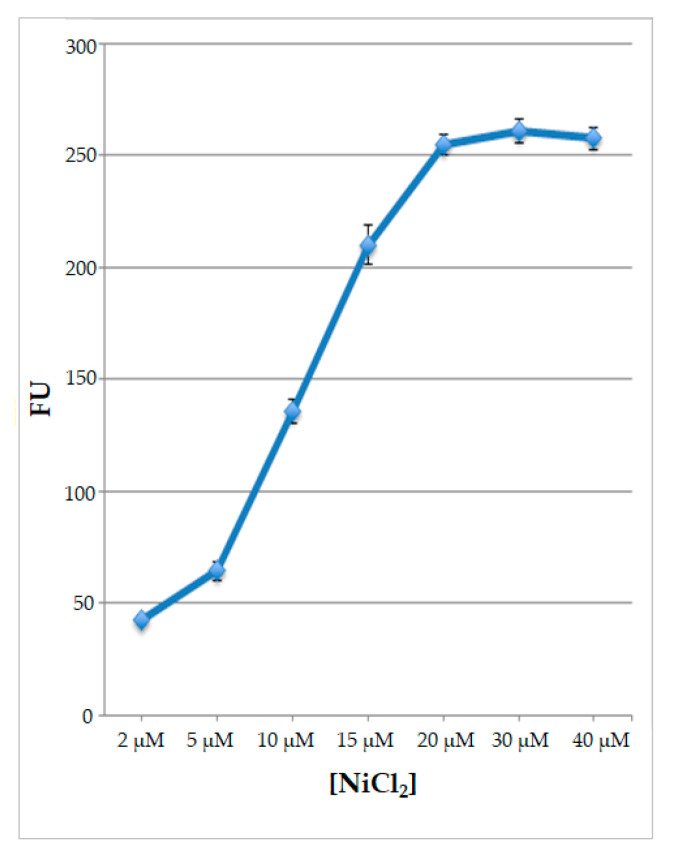
Values of fluorescence, expressed as fluorescence units (FUs) of protoplasts transformed with pPr*HSP17.6aGFP* plasmids and treated with different NiCl_2_ concentrations. Each value represents the mean of three independent measurements ± SD.

Since all the data indicated that nickel ions are the best inducers of fluorescence, to better characterize the response of the engineered protoplasts to this type of treatment, they were subjected for 2 h to various nickel ion concentrations, ranging from 2 μM to 40 μM. The results obtained indicate that, with rising Ni ion concentrations, the signal increased, reaching its maximum value at a concentration of 20 μM NiCl_2_; thereafter, the signal reached a plateau using higher Ni ion concentrations (Figure 7).

### 2.3. Nickel Ions Detection in Different Food Matrices

**Figure 8:**
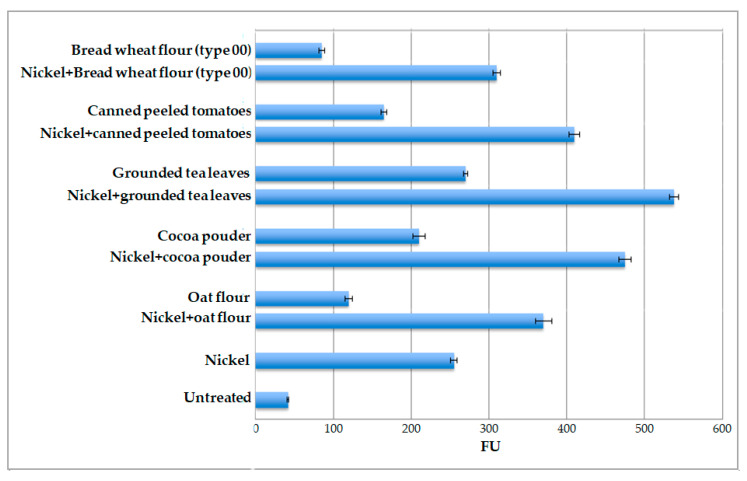
Values of fluorescence as expressed in fluorescence units (FUs) of protoplasts transformed with pPr*HSP17.6aGFP* plasmid plus the addition of different food matrices (in the presence or absence of 20 μM nickel ions) for 2 h. Protoplasts transformed with pPr*HSP17.6aGFP* plasmid with only nickel ions added (Nickel) and untreated protoplasts (Untreated) were used as controls. Each value represents the mean of three independent measurements ± SD.

Considering that nickel ions appear to be the most efficient in terms of eliciting fluorescence signals and that they are naturally present in many food matrices, the ability of the whole-cell system to detect Ni ions in food was tested. For this, the pPr*HSP17.6aGFP*-engineered protoplasts were tested against various food matrices, known to be “high-nickel foods” (canned peeled tomatoes, cocoa powder, ground tea leaves, oat flour) or “low-nickel foods”, namely bread wheat flour type 00, which is the most refined bread wheat flour [15,16,17]. All the food matrices were tested after adding exogenous nickel ions. A NiCl_2_ solution was added to reach a final concentration of 20 μM. The results, reported in Figure 8, indicate that the level of fluorescence signal is higher than that measured after treatment with nickel ions only, depending on the matrix utilized. The highest signal was obtained with ground tea leaves (FU 538), while the lowest signal was obtained with bread wheat flour type 00 (FU 310).

Subsequently, protoplasts were tested against the different food matrices only. In general, all food matrices are able to induce an increase in GFP expression. The results obtained, reported in Figure 8, confirm that ground tea leaves are the most effective inducers (FU 270), while bread wheat flour (type 00) is the least effective inducer (FU 85). In particular, for ground tea leaves, the level of induction was 6-fold higher than that obtained in untreated protoplasts, for cocoa powder it was 5-fold, for canned peeled tomatoes it was 4-fold, for oat flour it was 3-fold, and for type 00 wheat flour it was only 2-fold with respect to the same control, which was represented by untreated transformed protoplasts.

## 3. Discussion

Technological processes and agrochemical treatments are the main forces responsible for the contamination of food products and for the reduction in food nutritional value. The development of safe and accurate analytical methodologies for water and food control is crucial for the detection, analysis, and diagnosis of a wide range of compounds affecting food quality and healthiness. Heavy metal ions are among the worst and most dangerous contaminants because they are widely present in the environment and also because they are easily transferred from soil and water to living organisms [3,18].

Plants exposed to adverse environmental conditions (biotic or abiotic stresses) have developed complex molecular mechanisms to protect cell homeostasis and minimize the potential damage caused by these stimuli [19]. In general, a plant’s stress response is based on the activation and/or inactivation of gene expression, rapidly triggered after the perception of the stress. The rapidity of the response is due to the presence of different stress-responsive *cis* elements in the promoter region of these genes [20]. The most important group of genes participating in stress responses is constituted by the *heat shock* gene family. These genes are not only activated by heat stress, but also by other stressful environmental conditions, such as the presence of heavy metal ions [21,22].

With the aim of testing the ability of the promoter region of a plant’s small *HSP* gene to sense the presence of heavy metal ions in different food matrices, *N. tabacum* protoplasts were transformed with a plasmid containing the *GFP* gene controlled by the promoter region of the sunflower *HaHSP17.6a* gene. This specific promoter was chosen on the basis of the characterization, in a previous study, of the *HaHSP17.6a* gene, reported to be inducible in sunflower seedlings by heat stress as well as by other stimuli, and in particular by the presence of heavy metal ions [22].

The data obtained in vitro using this transient expression system established that the promoter region of *HaHSP17.6a* gene is activated by all the metal ions tested, although at different levels, confirming its inducibility, already demonstrated in vivo in sunflower seedlings [22]; the data also indicated that the *HaHSP17.6a* promoter exhibits the best sensitivity to Ni ions.

Having assessed promoter inducibility, the engineered protoplast system was also tested for its ability to sense the presence of heavy metal ions, with particular regard to Ni, in different food matrices. Systemic nickel allergy syndrome affects a significant portion of the population. Developing a more sensitive and effective tool for the rapid and in situ detection of high concentrations of nickel ions in food could be crucial. Such an advancement would play an important role in improving future food analysis. The food matrices utilized in this work were chosen mostly on the basis of their already known nickel content, according to their classification or as “high-Ni foods” or as “low-Ni foods” [15,17,23,24,25].

The first step of this part of this study was aimed at verifying whether the molecular composition of food matrices can “quench” nickel ions present in a solution. In order to carry that out, the determination of fluorescence emissions was performed via the addition of a known quantity of NiCl_2_ to the different matrices. Subsequently, the same tests were repeated using the food matrix alone. In both cases, the induction of fluorescence was detectable, and it specifically varied according to the food matrix assayed, indicating that none of the food matrices utilized had a molecular structure that could interfere with the biological detection of nickel ions present in the test solution.

In conclusion, taken together, the data obtained indicate that the engineered immobilized protoplast system established is a useful tool for detecting the presence of nickel ions in food. To our knowledge, this is so far the first example of using biosensors to detect traces of heavy metal ions in food based on genetically engineered plant protoplasts. Starting from these data, broader use of the biosensor realized can be hypothesized. One application could be its use to examine and evaluate a wider number of food matrices for the presence of nickel ions, as well as the use of this device for the detection of other heavy metal (Al, Cd, Co, Cu, Zn) traces as contaminants along the food chain. On the basis of these considerations, whole-cell biosensors appear to be the most suitable detection tool, not only because they are chips and are portable, but also because they are specifically designed to have high sensitivity in detecting heavy metal ions at trace levels. In other words, whole-cell biosensors can be considered the best way to measure heavy metal ions in food, thus contributing to providing larger benefits to consumer health in relation to food production and safety.

## 4. Materials and Methods

### 4.1. Plasmids Construction

A DNA fragment, corresponding to the promoter region of the sunflower small *HSP17.6a* gene [22], was amplified using the primers For-tgcctcgaggtagtacacggtg and Rev-gtaaaattgttcaacgtgttctagaggat. The primers contained restriction sites for generating *Xho*I and *Bam*HI ends. PCR was performed on genomic DNA obtained from sunflower seedlings (*Heliantus annuus* L., cv. Gloriasol) using “Pure Link Plant Total DNA purification” kit (Invitrogen, Carlsbad, CA, USA), according to supplier’s instructions. In order to verify an amplicon’s identity, the PCR product was purified and sequenced using standard procedures; the DNA sequence, 1769 bp long, was compared with the corresponding genomic clone (accession number AJ306557.2). The DNA fragment obtained was cloned into a vector containing the *GFP* (green fluorescent protein) gene, followed by *nopaline synthase* gene (*Nos*) terminator, previously digested with *Xho*I and *Bam*HI; the plasmid obtained was named pPr*HSP17.6aGFP*. A plasmid containing the *GFP* gene under the control of *CAMV35S* promoter*,* named p*35SGFP*, was used as the control.

### 4.2. Protoplasts Preparation, Transformation and Immobilization

*N. tabacum* cultivar SR1 protoplasts were prepared from leaves of 7–8-week-old plants. The transformation was performed using PEG-mediated direct gene transfer, as previously described by Gullì and coworkers [26]. Ten micrograms of each constructed plasmid were used for the transformation of ~600,000 protoplasts. The protoplast suspension was gently mixed with a solution of 0.6% agarose in a K3 medium [27] at 40 °C; 150 μL of the mixture was distributed in a multiwall plate and solidification was completed after 30 min of treatment at room temperature. Immobilized protoplasts were kept at room temperature until stress treatments were performed.

### 4.3. Protoplasts Stress Treatments with Different Heavy Metal Ions

Transformed protoplasts were subjected to metal ion stress by adding 50 μL of 20 μM of AlCl_3_, CdSO_4_, CoCl_2_, and ZnSO_4_, or 2 μM, 5 μM, 10 μM, 15 μM, 20 μM, 30 μM, and 40 μM of NiCl_2,_ at room temperature. They were observed after 1, 2, 3 and 4 h for fluorescence determination. The controls used were wild-type protoplasts (WT) and protoplasts transformed with the p*35SGFP* plasmid and then subjected to stress. We also used WT protoplasts and protoplasts transformed with the p*35SGFP* plasmid and pPr*HSP17.6aGFP* but not subjected to stress.

### 4.4. FDA Assay for Protoplast Viability Estimation

Fluorescein diacetate (FDA) staining was used to determine the viability of protoplasts. Fifty µL of the K3 medium, containing untransformed or transformed protoplasts, was transferred into a microtube; 1 µL of 0.2% FDA solution, dissolved in acetone, was added and incubated at room temperature for 2 min. All FDA-treated protoplasts were observed in a K3 culture medium and after agarose (0.6%) immobilization. Only viable protoplasts appeared to be green and fluorescent under the confocal laser microscope (LSM 710, Carl Zeiss, Oberkochen, Germany). For the viability measurements, three images were selected for each sample; the percentage of protoplast viability was expressed as the ratio between the number of the fluorescent protoplasts and the total number of protoplasts ×100. Each experiment was repeated three times.

### 4.5. Confocal Microscopy and Fluorescence Determination

For protoplast observation, a laser scanning confocal microscope (LSM 710, Zeiss) was used. To control protoplast viability after transformation and/or immobilization, protoplasts were observed in their culture medium and after agarose immobilization. To detect GFP or FDA fluorescence, a 488 nm argon ion laser line was used, and the emission was recorded with a 505–530 nm filter set. Chlorophyll epifluorescence was detected with the filter >650 nm and eliminated after He-Ne laser excitation at 543 nm, as previously reported [28]. The power of each laser line, the gain, and the offset were identical for each experiment so that the images were comparable. For fluorescence quantification, the Profile Tool of the ZEN2012 program of the LSM 710 confocal microscope was used. The mean of pixel intensities relative to the GFP channel was used for fluorescence quantification; 20 protoplasts were measured for each treatment to produce the quantification analysis and three independent experiments were performed. Images were processed using Adobe Photoshop 7.0 software (Mountain View, CA, USA). Protoplast fluorescence was also measured using and Infinite F200 fluorometer (TECAN, Männedorf, Switzerland) set as follows: excitation at 485 nm (±20) and emission at 510–560 nm. Fluorescence values are presented as arbitrary fluorescence units (FUs) or as relative fluorescence unit (RFUs), namely, the ratio of the fluorescence of the treated sample to that of the untreated control (response ratio). All data are the mean ± SD of three different measurements.

### 4.6. Food Matrices Utilized for Nickel Ions Detection

The food matrices utilized to assess the responsiveness of engineered protoplasts to the presence of metal ions in food were oat flour, cocoa powder, ground tea leaves, canned peeled tomatoes, and bread wheat flour (type 00). We added two grams of each food matrix to 8 mL of sterile distilled water and stirred the mixture for 2 h. One mL was centrifuged at 13,000 rpm for 2 min; 50 μL of supernatant, without or with NiCl_2_ at the final concentration of 20 μM, was added to immobilized protoplasts.

### 4.7. Statistical Analysis

Statistical analysis was performed using the SigmaStat version 3.11 software (Systat Software Inc., Chicago, IL, USA) as appropriate. The viability of protoplasts floating in K3 buffer or immobilized in 0.6% agarose were compared using Student’s *t*-test. The protoplast fluorescence values, measured fluorometrically or via confocal microscope tools, were analyzed via the pairwise multiple comparisons procedure using the Holm–Sidak method. All values were expressed as means ± standard deviation (SD) of at least three independent replicated experiments (n = 3). A *p* value ≤ 0.05 was considered to be statistically significant.

## Figures and Tables

**Table 1:** Protoplast viability (%), determined by FDA assay, of untransformed and p*35SGFP-* and pPr*HSP17.6aGFP*-transformed protoplasts floating in a K3 medium or immobilized in K3 medium containing 0.6% agarose.

	Floating Protoplasts (%)	Immobilized Protoplasts (%)
WT	98.77 ± 1.23	97.70 ± 2.30
p*35SGFP*	99.05 ± 0.95	98.44 ± 1.56
pPr*HSP17.6aGFP*	97.87 ± 2.13	98.65 ± 1.35

Each value represents the mean of three independent measurements ± SD. WT, untrasformed protoplasts; p*35SGFP*, protoplasts transformed with p*35SGFP* plasmid; pPr*HSP17.6aGFP*, protoplasts transformed with pPr*HSP17.6aGFP* plasmid.

**Table 2:** Fluorescence index given by the mean of GFP pixels intensity per protoplast transformed with pPr*HSP17.6aGFP* and treated with the different metal ions (50 μL of 20 μM each). Differences between NiCl_2_-treated protoplasts and each different metal ion were significant (*, *p* < 0.05) or highly significant (**, *p* < 0.001).

	Mean ± SD of GFP Pixel Intensity per Protoplast
AlCl_3_ (2 h)	4.76 ± 0.43 **
CdSO_4_ (2 h)	4.21 ± 0.46 **
CoCl_2_ (2 h)	5.27 ± 0.66 **
CuSO_4_ (3 h)	4.02 ± 0.51 **
NiCl_2_ (2 h)	7.30 ± 0.46
ZnSO_4_ (2 h)	5.18 ± 0.52 *

## Data Availability

The original contributions presented in this study are included in the article.
